# A simple method for calculating minimum estimates of previous population sizes of wildlife from hunting records

**DOI:** 10.1371/journal.pone.0198794

**Published:** 2018-06-12

**Authors:** Naoki Agetsuma

**Affiliations:** Tomakomai Experimental Forest, Field Science Center for Northern Biosphere, Hokkaido University, Tomakomai, Hokkaido, Japan; Universitat Autonoma de Barcelona, SPAIN

## Abstract

Hunting records have proven useful for examining the historical status of wildlife populations. The number of animals harvested can provide information on past population sizes that would have been required to support harvest yields. Therefore, when statistical data on annual harvests are available, a minimum estimate of past population sizes can be calculated. A very simple method for estimating the sizes of historic wildlife populations using only annual hunting records and the maximum annual population increase rate is presented in this study. This method was applied to estimate past population sizes for Japanese sika deer (*Cervus nippon yesoensis*) in Hokkaido Island, Japan, using hunting records from 1873 to 1882, and assuming 15% and 35% population increase rates. The annual number of deer harvested during 1873 to 1882 ranged from 15,000 to 129,000. The minimum population size in 1873 was estimated as 349,000–473,000. This method was validated by applying it to the eastern population of Hokkaido Island in 1993 when the population size was approximately 260,000, and population sizes estimated by this method were 0.50–1.17 times the nominal population size. Thus, the population estimates from this method were approximately equal to or less than the expected population sizes, and this method can be used to obtain minimum estimates of wildlife populations. Because shorter durations of hunting records result in population size underestimates, it would be better to use hunting record of 10 years or longer in this method. In addition, the degree of underestimation may change with hunting pressure intensity on the populations, other causes of mortality, and maximum annual increase rates of the species. The method can be applied to any wildlife species for which records of annual harvest and maximum annual population increase rates of the species are available. The estimates obtained can provide benchmarks for the population size required for ecosystem conservation, and can be useful for wildlife management as they indicate the lowest limit to maintain the population.

## Introduction

Wildlife population size has been estimated using many models that require information on various population parameters such as carrying capacity (for logistic growth models), age structure, age-specific survival and reproduction rates (for age structure models), demographic and environmental stochasticities (for logistic growth models and age structure models), catch-per-unit-effort (CPUE), and catch-effort (CE) (for Poisson catchability models) [1–3]. However, obtaining or estimating these parameters would be difficult or impossible for past populations. Therefore, the models requiring multiple population parameters as inputs can only be applied to the populations for which detailed ecological information is available. Models that use easily obtainable parameters, and require a small number of assumptions would have a much wider application for the estimation of past populations.

Hunting records (number of individuals harvested) have proven useful for examining the past status of wildlife populations [4,5]. A famous example by MacLulich [4] involved the reconstruction of population dynamics of lynx (*Lynx canadensis*) and hare (*Lepus americanus*) based on fur trade statistics although he did not try to estimate the actual population sizes of these species; the number of traded furs was assumed to reflect the relative population sizes. Hunting records can also provide information on the past population sizes that would have been required to support harvest yields. When statistical data on past annual harvests are available, a minimum estimate of population sizes can be calculated for the corresponding period. Here, I propose a very simple method for obtaining a minimum estimate of population sizes for historic wildlife populations from the annual number of individuals harvested.

Estimates of historic wildlife population sizes are important for current initiatives of ecosystem conservation and wildlife management. These can indicate the minimum size of wildlife population that must be maintained for a “sound” natural ecosystem. In addition, they may also provide a basis for determining when populations are overabundant and need to be controlled. For example, many researchers regarded the populations of ungulates as overabundant simply on the basis of recent changes in vegetation structure [6–8]. However, they did not consider population sizes of ungulates in less degraded ecosystem or more natural ecosystem in the past, which should be an important measure for judging the degree of overabundance and the degree of degradation of the current ecosystem, as some wild animals like ungulates could be indicators of environmental integrity. Thus, past population sizes, even the minimum estimates, can constitute the bases for judging overabundance of present populations.

In Japan, intensive forestry activity from the 1960s to the 1970s resulted in the destruction of habitats and extensive impacts on wildlife populations [9]. Therefore, for wildlife management and ecosystem conservation, it is important to know the sizes of wild populations before significant habitat disturbances occurred. In the present study, a simple model has been proposed to calculate minimum estimates of historic wildlife population sizes using only annual hunting records and the maximum annual population increase rate of the species. This model is applied to estimate the population size of one of the subspecies of Japanese sika deer (Ezo sika deer, *Cervus nippon yesoensis*) inhabiting Hokkaido Island, using records of annual hunt yields from 1873 to 1882. The model proposed in this study was validated by applying it to a deer population in the eastern part of Hokkaido Island in 1993, for which the population size was estimated by a conventional method.

## Methods

### Ethics statement

I did not involve direct contact with any animals because I analyzed the data in literature in this study.

### Subject

The Ezo sika deer is an endemic subspecies of Hokkaido Island (ca. 78,000 km^2^) in northern Japan. Before the 1870s, a large population of deer inhabited Hokkaido [10], despite the presence of wolves (*Canis lupus*), before becoming extinct around 1900 [11]. The heavy snowfall in 1879, over-hunting, and exploitation of natural environment are believed to be the factors responsible for the drastic decrease in the deer population in Hokkaido [10,12], leading to its extinction in various areas of the island thereafter. However, the deer population started to recover from its decline in the 1980s, probably because of improvement in habitat conditions concomitant with the decline of the forestry industry, and recovery of vegetation and expanses of pastures. Owing to the population recovery, the estimated population size of deer between 2013 and 2016 was around 500,000–600,000 individuals (data of Hokkaido Prefecture in 2017). Hokkaido Prefecture [13] considered that the deer population was too large and developed a management plan to reduce the eastern population to 5–25% of its current size to prevent agricultural damage and to conserve the natural ecosystem. Hokkaido Prefecture intensified the hunting pressure since 1998, and more than 100,000 deer are being culled annually through pest control or hunting after 2009 in Hokkaido Island [13].

Currently, approximately 70% of the area of Hokkaido Island is covered by forests and natural vegetation (data from Ministry of Land, Infrastructure, Transport and Tourism). Deer inhabit these areas as well as the land used for agriculture. As most part of the island is inhabited by deer, the total habitat area recovered to nearly its original extent, although habitat quality may have changed by altering the exploitation intensity of the natural environment after the 1870s.

### Estimation of past population

The population size in year t (P_t_) was obtained by subtracting the population size in year t-1 (P_t-1_) from the number of individuals harvested in year t-1 (H_t-1_), and multiplying it by the rate of population increase (r) plus 1 (Eq 1).

$$P_{t}=(P_{t-1}-H_{t-1})\times (r+1)$$ Eq 1

Eq 1 is transformed into Eq 2.

$$P_{t-1}=H_{t-1}+P_{t}/(r+1)$$ Eq 2

The population size can then be estimated backward in a sequential manner. However, the population size in year t (P_t_) is not available in many cases. In such cases, the number of individuals harvested in year t (H_t_) is used as the population size. Then, P_t-1_ can be estimated from Eq 2. It should, however, be noted that unless the population was extirpated by hunting in year t, P_t_ and P_t-1_ should be underestimated. Consequently, subscripts in Eq 2 are changed from t to t-1 and from t-1 to t-2, and the population size in the year t-2 (P_t-2_) is obtained from the estimated value of P_t-1_ and the number of individuals harvested in year t-2 (H_t-2_). By iteration, the population size can be estimated backwards from the most recent year to the first year of the period for which consecutive hunting records are available.

Use of Eq 2 to estimate the past population size requires the past annual rate of population increase (r), which is commonly difficult to obtain. Therefore, the maximum annual rate of increase (intrinsic population growth rate) of the species is used in place of “r”. Since the wildlife populations do not realize their maximum annual rate of population increase every year, the estimated population size will be an underestimate. For Japanese deer, a maximum annual rate of population increases of 15–19% has been generally applied [14,15]. Although, the deer populations may increase by more than 19% annually under some conditions. For example, a population of Japanese sika deer (*C*. *n*. *centralis*) on Kinkazan Island (ca. 10 km^2^) increased by 35.9% between 1984 and 1985 following their mass mortality [9,16]. As the Japanese sika deer populations may have the potential to increase by around 35% in a year, two maximum annual population increase rates, 15% and 35%, were used in this study.

### Hunting records

**Fig 1:**
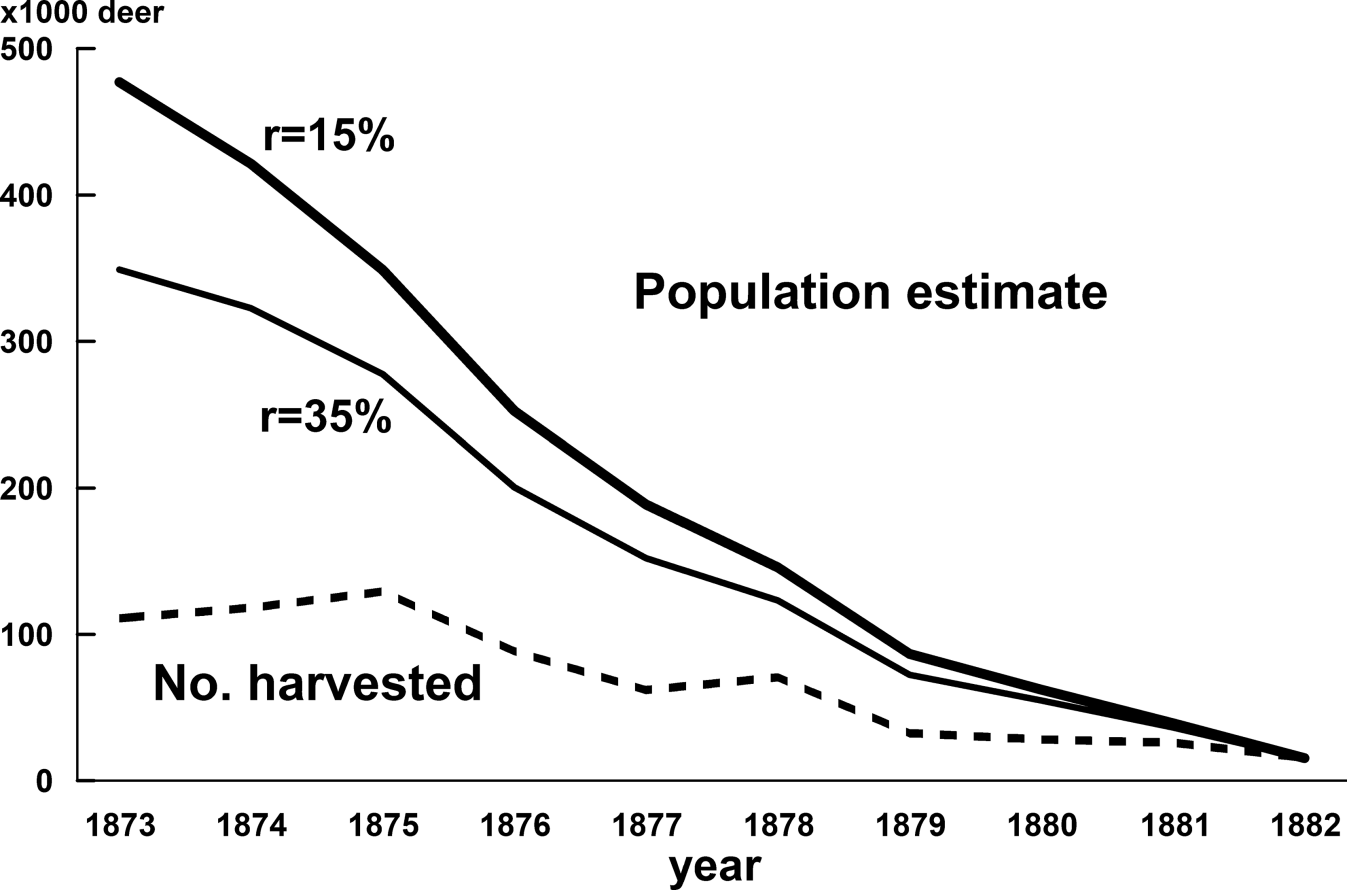
Annual number of deer harvested (dashed line) and population estimates (solid lines) in Hokkaido Island from 1873 to 1882 by Eq 2 based on annual population increase rates (r) of 15% and 35%, respectively.

**Fig 2:**
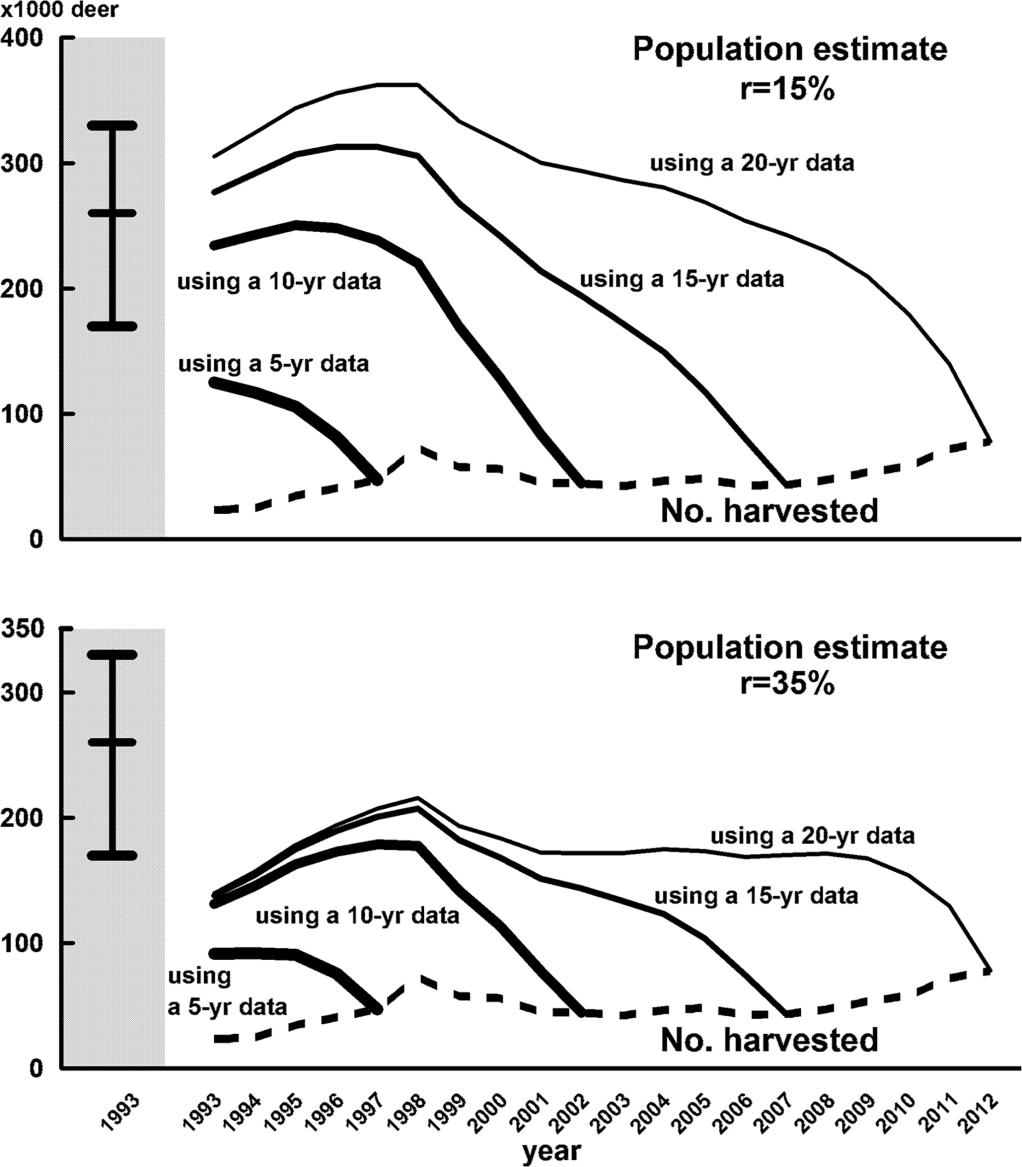
Annual number of deer harvested (dashed lines) and estimates (solid lines) of the eastern population of Hokkaido Island from 1993 to 2012 by Eq 2 based on annual population increase rates (r) of 15% (upper) and 35% (lower), respectively. A vertical bar indicates the population size in 1993 estimated by [13] and [20].

Hunting records of Ezo sika deer in Hokkaido Island over a period of ten years, from 1873 to 1882 (15,429–129,166 deer/year; Fig 1, S1 Dataset) [10,17], can be used to estimate the population size in 1873 using Eq 2. In addition, for the purpose of validation, the model proposed in this study was applied to deer population in the eastern part of Hokkaido Island in 1993 which was designated as the base year of the deer population management by Hokkaido Prefecture [13]. The eastern population of deer could be distinguished from other local populations by their mitochondrial type [18]. Similar to the estimation of the deer population in 1873, a 10 year hunting record (from 1993 to 2002) [19] was used for the estimation of population size in 1993. In addition, to test the effect of the length of the hunting dataset used in the proposed model, a hunting record for periods of 5 years (from 1993 to 1997), 15 years (from 1993 to 2007) and 20 years (from 1993 to 2012; Fig 2, S1 Dataset) [13,19] were also used. The population size in the year 1993 was approximately 260,000 [13], and the 95% confidence interval estimated by hunting records and demographic parameters of doe, buck, and fawn ranged 170,000–330,000 [20].

## Results

The minimum estimates of the population size of Ezo sika deer between 1873 and 1882 were calculated using Eq 2 with r-values of 15% and 35% (Fig 1, S2 Dataset). Back-calculation of the population size from 1882 to 1873 in a sequential manner showed that the estimated population of deer increased uniformly. The estimated numbers of deer in 1873 were 473,312 and 349,044 when the r-values were assumed to be 15% and 35%, respectively.

For validation of the model, the size of the eastern population of Hokkaido Island in 1993 was also estimated by Eq 2 (Fig 2, S3 Dataset). Using hunting records over a period of 10 years and r-value of 15%, the minimum estimate of population size in 1993 was 234,372, which corresponded to 0.90 (0.71–1.38) times the population size estimated by other methods for the same year (260,000 [13]; 95% confidence interval 170,000–330,000 [20]). Using the r-value of 35%, the estimated population size was 131,194, which was 0.50 (0.40–0.77) times the population size estimated by other methods. Using hunting records for 5, 15, and 20 years and r-value of 15% in the proposed model, the estimate of population sizes were 0.48 (0.38–0.74), 1.06 (0.84–1.63), and 1.17 (0.92–1.79) times of the population size estimated in 1993 by other methods, respectively (Fig 2, S3 Dataset). For the r-value of 35%, the estimates using hunting records for 5, 15, and 20 years corresponded to 0.35 (0.28–0.54), 0.53 (0.40–0.77), and 0.54 (0.42–0.82) times lower than the reference population size for the year 1993, respectively. Differences of all estimates using 10- and 15-year data from those using a 20-year data were within 23%; however, the differences of estimates using a 5-year data were 34% (r = 35%) and 59% (r = 15%).

## Discussion

Similar to other models for the estimation of wildlife population sizes using hunting records, the model proposed in this study assumes that the number of harvested wildlife is correctly recorded [e.g. 2,20]. If there are many omissions of hunting reports and/or illegal hunting, the population will be underestimated by the method proposed. However, to obtain a minimum estimate of population size, amplification of hunting reports is a great concern that leads to overestimation, although it is unlikely to occur unless fraudulent rewards are claimed for killing wild animals. It should also be noted that the reported number of hunted animals is usually lower than the actual number of hunting-related mortalities because it does not include injured animals that escaped and died later. For white-tailed deer (*Odocoileus virginianus*), such cases may account for more than 30% of the total number of harvested [21].

The number of individuals harvested in the latest year was used as the population size for the estimation procedure. However, it is not probable in most cases that all animals were extirpated by only hunting in the latest year. If some of the animals died by other causes in the year and/or could survive after the year, the population size must be underestimate. The influence works strongly within a shorter time period from the latest year of the analysis. Population estimates in 1993 using 5, 10, 15, and 20-year data showed that the differences among the estimates greatly decreased as the duration of the hunting records increased (Fig 2). Thus, it would be better to use hunting records of 10 years or longer for the application of this model. If the population size estimated by other methods is available in the latest year, it can be used as the initial population size (Pt) in Eq 2. This will solve the problem relating to the length of hunting records.

When the harvest yields are relatively small for wildlife population sizes because of social and cultural factors, the population estimated by this model will be much smaller compared to the actual population sizes. Thus, in this model, population estimates on the basis of hunting records would commonly lead to underestimation. This model also assumes that hunting is the only factor restricting the growth of wildlife populations, and the maximum annual rate of population increase for the species was used as the rate of increase in each year. However, wild populations are usually not expected to increase at the maximum possible rate every year, and factors other than hunting, such as food resources, population density, weather, predators and disease also have a negative impact on growth rates [22–25]. For example, if population sizes of wildlife are also restricted by food resources (i.e., carrying capacity), as assumed by logistic growth models [2], the annual rate of increase should be far lower than the maximum annual growth rate when the populations come close to the capacity. If wild populations are indeed restricted by such factors, and cannot attain their maximum annual rate after hunting, population sizes larger than the estimates should be necessary to achieve the reported numbers of deer harvested annually. The influence of these factors may be amplified in species with very high maximum annual growth rates (e.g. voles [26]). Because possible gaps between the maximum growth rates and actual annual increase rates could become greater in those species, the gaps raise the degrees of underestimation. For wildlife showing boom-bust population cycles (e.g. [4]), the gaps will become greater during decreasing periods than increasing periods of the populations. However, if past population increase rates are uncertain, it is prudent to use the maximum annual rate of population increase of the species to obtain the minimum estimates. Because, if actual population growth frequently exceeds the assumed rate, the population estimates might be overestimated.

As mentioned above, the degree of underestimation in population estimates in this model depends on hunting pressures on the population, other causes of mortality than hunting, length of hunting records, and maximum annual increase rates of the species. For applications of the model to wild populations, these conditions should be considered.

The proposed model was applied to the eastern population of Hokkaido Island in 1993. The population estimates were similar to or less than the population size estimated by other methods (Fig 2). This suggested that the model used in this study tends to underestimate the population size, as expected. Thus, this model is valid to calculate the minimum estimates of wildlife populations. If the degree of underestimation in 1993, using the 10 years’ hunting records, was the same as that in 1873, the actual population sizes in 1873 were 525,000 (343,000–666,000: r = 15%) and 692,000 (452,000–878,000: r = 35%).

More plausible estimates of population sizes can be obtained using conventional models when detailed ecological information (e.g. carrying capacity, CPUE, environmental stochasticity, age structure, and age-specific survival and reproduction rates [1–3,20]) is available. However, when attempting to estimate past population sizes, data for the required parameters are not available and assumptions of the models cannot be verified in many cases. Therefore, such conventional methods can be applied only to limited populations for which additional information is available. On the other hand, the method described in this study requires only two parameters (annual number of animals harvested, and the maximum annual rate of population increase of the species). This simplicity makes this method applicable to many previous wildlife populations. Further studies are required to evaluate the extent to which this method underestimates the past population. The method proposed in this study and conventional methods can be applied to the same populations for which detailed ecological information is available, and comparison of the results can reveal the validity of the method, as in the present study.

Although this simple method estimates only the minimum population size of wildlife in the past, it is important for wildlife management. The estimates can provide a benchmark for the population size required for ecosystem conservation or the lowest limit for population control of wildlife. This information is important for the management of wild populations but is difficult to determine using other methods. The population size of Ezo sika deer in Hokkaido Island between 2013 and 2016 (500,000–600,000) cannot be significantly larger than the minimum population size estimates for 1873 when the underestimation is taken into account. This suggests that the recent deer population cannot simply be regarded as overabundant relative to that in the past condition of Hokkaido Island, although we should consider the carrying capacities of the ecosystem for deer in the past and present. For formulating wildlife management plans, it is important to note that the figures calculated by this method are underestimates of the population size, and the actual past population size might have been much greater than the estimates.

## Supporting information

S1 Dataset Annual number of deer harvested in Hokkaido Island from 1873 to 1882 and that in the eastern part of Hokkaido Island from 1993 to 2012 in Fig 1 and Fig 2. (CSV)

S2 Dataset Population estimates of deer in Hokkaido Island from 1873 to 1882 by Eq 2 based on annual population increase rates (r) of 15% and 35%, respectively in Fig 1. (CSV)

S3 Dataset Population estimates of deer in the eastern part of Hokkaido Island from 1993 to 2012 by Eq 2 based on annual population increase rates (r) of 15% and 35%, respectively in Fig 2. (CSV)

## References

[1] Sæther B-E, Engen S, Islam A, McCleery R, Perrins C. Environmental stochasticity and extinction risk in a population of a small songbird, the great tit. Am Nat. 1998;151(5): 441–450. doi: 10.1086/286131 18811318 10.1086/286131

[2] Skalski JR, Ryding KE, Millspaugh J. Wildlife Demography: Analysis of Sex, Age, and Count Data Boston: Elsevier Academic Press; 2005. 636p.

[3] Saltz D, Rubenstein DI, White GC. The impact of increased environmental stochasticity due to climate change on the dynamics of Asiatic wild ass. Conserv Biol. 2006;20(5): 1402–1409. doi: 10.1111/j.1523-1739.2006.00486.x 17002758 10.1111/j.1523-1739.2006.00486.x

[4] MacLulich DA. Fluctuations in the numbers of the varying hare (*Lepus americanus*). University of Toronto Studies, Biological Series. 1973;43: 1–136.

[5] Kay CE. Are ecosystems structured from the top-down or bottom-up: a new look at an old debate. Wildl Soc Bull. 1998;26(3): 484–498.

[6] Augustine DJ, de Calesta D. Defining deer overabundance and threats to forest communities: From individual plants to landscape structure. Ecoscience. 2003;10: 472–486.

[7] Suzuki M, Miyashita T, Kabaya H, Ochiai K, Asada M, Tange T. Deer density affects ground-layer vegetation differently in conifer plantations and hardwood forests on the Boso Peninsula, Japan. Ecol Res. 2008;23(1): 151–158.

[8] Hurley PM, Webster CR, Flaspohler DJ, Parker GR. Untangling the landscape of deer overabundance: Reserve size versus landscape context in the agricultural Midwest. Biol Conserv. 2012;146: 62–72.

[9] Agetsuma N. Ecological function losses caused by monotonous land use induce crop raiding by wildlife on the island of Yakushima, southern Japan. Ecol Res. 2007;22(3): 390–402.

[10] Inukai T. The sika deer in Hokkaido and its rise and decline. Rep Northern Cult Res. 1952;7: 1–45. (in Japanese)

[11] Umeki K. Research history of extinct wolves in Hokkaido: Overview and issue. Res J Grad Students Lett. 2015;15: 35–67. (in Japanese)

[12] Kaji K, Miyaki M, Uno H. Conservation and management of sika deer in Hokkaido Sapporo: Hokkaido University Press; 2006. 247p. (In Japanese)

[13] Prefecture Hokkaido. Ezo sika deer management plan Sapporo: Hokkaido Prefecture; 2017 25p. (in Japanese) Available from: http://www.pref.hokkaido.lg.jp/ks/est/ezosikakannrikeikaku.htm

[14] Kaji K, Okada H, Yamanaka M, Matsuda H, Yabe T. Irruption of a colonizing sika deer population. J Wildl Manage. 2004;68(4): 889–899.

[15] Matsuda H, Kaji K, Uno H, Hirakawa H, Saitoh T. A management policy for sika deer based on sex-specific hunting. Res Popul Ecol. 1999;41(2): 139–149.

[16] Japan Nature Conservation Society. Wildlife conservation Tokyo: Nature Conservation Society of Japan; 1991. 320p. (in Japanese)

[17] Hokkaido Prefecture. Report on animal distributions Sapporo: Hokkaido Nature Preservation Division, Hokkaido Prefecture; 1987. 100p. (In Japanese)

[18] Ou W, Takekawa S, Yamada T., Terada C, Uno H, Nagata J, et al Temporal change in the spatial genetic structure of a sika deer population with an expanding distribution range over a 15-year period. Popul Ecol. 2014;56(2): 311–325.

[19] Hokkaido Prefecture. Review of Ezo sika deer conservation plan Sapporo: Hokkaido Prefecture; 2007 24p. (in Japanese) Available from: http://www.pref.hokkaido.lg.jp/ks/skn/sika/sikatop.htm

[20] Matsuda H, Uno H, Tamada K, Kaji K, Saitoh T, Hirakawa H, et al Harvest-based estimation of population size for sika deer on Hokkaido Island, Japan. Wildl Soc Bull. 2002;30: 1160–1171.

[21] Roseberry JL, Autry DC, Klimstra WD, Mehrhoff LA Jr. A controlled deer hunt on Crab Orchard National Wildlife Refuge. J Wildl Manage. 1969;33: 791–795.

[22] Skogland T. What are the effects of predators on large ungulate populations? Oikos. 1991;61: 401–11.

[23] Fischer JR, Hansen LP, Turk JR, Miller MA, Fales WH, Gosser HS. An epizootic of hemorrhagic disease in white-tailed deer (*Odocoileus virginianus*) in Missouri: necropsy findings and population impact. J Wildl Dis. 1995;31(1): 30–36. doi: 10.7589/0090-3558-31.1.30 7563421 10.7589/0090-3558-31.1.30

[24] Hodges KE, Krebs CJ, Hik DS, Stefan CI, Gillis EA, Doyle CE. Snowshoe hare demography In: Krebs CJ, Boutin S, Boonstra R, editors. Ecosystem dynamics of the boreal forest. New York: Oxford University Press; 2001 pp. 141–178.

[25] Sibly RM, Hone J, Clutton-Brock TH. Wildlife population growth rates Cambridge: Cambridge University Press; 2003. 362p.

[26] Turchin P, Ostfeld RS. Effects of density and season on the population rate of change in the meadow vole. Oikos. 1997;78: 355–361.

